# Health benefits of cycle ergometer training for older adults over 70: a review

**DOI:** 10.1186/s11556-015-0152-9

**Published:** 2015-11-02

**Authors:** Walid Bouaziz, Elise Schmitt, Georges Kaltenbach, Bernard Geny, Thomas Vogel

**Affiliations:** Geriatric Department, University Hospital, Strasbourg, France; Department of Physiology and EA-3072, Faculty of Medicine, Strasbourg University, Strasbourg, France; Functional Explorations Department, University Hospital, Strasbourg, France

**Keywords:** Health benefits, Cycle ergometer training, Older adults, Over 70

## Abstract

As the number of older adults continues to increase worldwide, more attention is being paid to geriatric health care needs, and successful ageing is becoming an important topic in the medical literature. A preventive approach to the care of older adults is thus a priority in our aging societies. The purpose of this study was to update evidence for the health benefits of cycle ergometer training for older adults over 70. We searched online electronic databases up to September 2014 for original observational and intervention studies on the relationship between cycle ergometer training and health among older patients over 70. Twenty-five studies examined interventions aimed specifically at promoting cycling for older adults over 70. These studies reported a positive effect on the prevention of cardiovascular disease, and a significant improvement in metabolic responses. Improving functional status, muscle strength and cognitive performance are also well established. Overall, this review demonstrates a positive effect of cycle ergometer training with functional benefits and positive health outcomes for older adults over 70. Based on this evidence, clinicians can now encourage older adults to profit from the health benefits of cycle ergometer training to be able to pursue their daily activities independently.

## Introduction

Physical activity (PA) is a modifiable behavior associated with health, functional status, and longevity, and encouraging a physically active lifestyle has become an accepted public health objective [1, 2].

Concerning the general population, it is well known that regular physical activity (PA) ensures primary and secondary prevention of several chronic conditions [3]. Indeed, a large scale longitudinal 8-year study found that every additional 15 min of daily PA up to 100 min per day resulted in a further 4 % decrease in mortality from any cause [4].

The prevalence of sedentary behavior is higher in older adults, and is an independent health risk factor lead to an increase in the risk of developing numerous chronic diseases as well as all-cause mortality [5, 6]. The risk of all of these diseases can be reduced by increased physical activity [7]. Further, evidence suggests that for older people, PA in later life can extend the period of active independent living, reduce disability and improve the quality of life [8]. Increasing PA thus helps minimize the burden on health and social care by improving healthy ageing [9, 10].

It is well established that endurance exercise training is an effective strategy in older adults to reduce body fat mass, increase whole-body insulin sensitivity, and reduce the risk of cardiovascular disease [3, 11]. It is also an effective strategy to protect older adults from falls and from some cancers (especially breast and colon cancer) [3].

The effectiveness of different modes of endurance exercise training on health such as cycle ergometer training (CET) [12], walking or jogging [13] and treadmill training [14] has been demonstrated in several studies. Among these modes of exercise, CET is particularly attractive because it is relatively easy and safe and causes no related injuries [15].

CET is usually a major component of any endurance exercise program that seeks to improve aerobic capacity and cardiovascular health [16]. Cycling is a healthy form of endurance exercise and, as a non-weight bearing activity, has less impact on the joints and is thus less stressful for the body than jogging or other running sports [17]. In addition, CET does not require as much postural control as walking on a treadmill and may be a better alternative for individuals with poor balance [18]. Finally, CET is feasible even for frail older individuals [19].

Up to now, the health benefits of CET for the general population are well established [20–22]. However, the effect of CET is less well documented in older adults [23–26] particularly those aged over 70 [27, 28]. The aim of this review was thus to assess the health benefits of CET in this group.

## Methods

### Literature search

We conducted a systematic search for observational and intervention studies that examined the relation between cycle exercise training and the health of older adults published between April 1983 and June 2013. Published and peer-reviewed articles in English language journals were identified in electronic databases and on reference lists of articles available to the authors of this review.

The search terms ‘exercise training, endurance training, or aerobic training and cycle ergometer, cycling or bicycle and health, health benefits and older adults, elderly, very old/elderly, aged, aging, oldest, old and over 70’ were combined to search each of the eight electronic databases. The search resulted in a total of 4080 hits: Pubmed Central 3245, Medline 269, Scopus 237, Web of Science 129, SportDiscus 62, Embase 56, CINAHL Plus 50, Cochrane library 32. We also searched for previous systematic reviews, websites, and references therein.

### Inclusion criteria and selection process

Based on the article titles and available abstracts, the reports were first evaluated for inclusion using the following criteria:

Original research articles written in English, observational or intervention studies published in peer-reviewed journals with outcomes based on the use of bicycle ergometer, independently reported effects of cycle ergometer training (CET) in adults aged over 70, quantitative measures of CET for any purpose, measures of mortality or morbidity (including disease risk factors) and/or measures of health and cardiovascular function through CET.

Altogether 3964 articles were excluded for the following reasons: absence of controls studies, the outcomes were based on the use of real cycling, the studies focused on cycling injuries and accidents, and the studies evaluated physical activity in general, and not specifically CET.

**Fig. 1:**
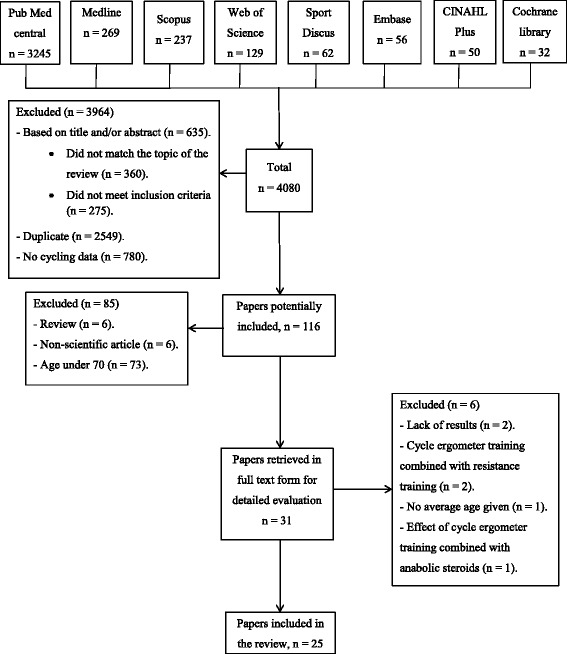
Flow chart of the literature search

A total of 116 potentially relevant papers were selected. Two authors independently evaluated them based on the inclusion criteria. This resulted in the additional exclusion of 85 studies. Thirty-one of the originally eligible studies were selected for detailed evaluation of the full text. After exclusion of six more papers based on the unanimous judgment of the two authors, 25 studies were selected for review (Fig. 1).

### Data extraction

The selected studies were sorted according to authors, year of publication, mean age of sample, type of protocol and outcomes. The studies were divided into four groups based on four outcomes: cardiovascular function (Table 1), metabolic outcomes (Table 2), functional status (Table 3), and cognitive function (Table 4).

**Table 1:** Summary of studies that analyze the effects of cycle ergometer training on cardiovascular function

			Outcomes		
			Cardiovascular function		
Author, year	Mean age (years)	Protocol type	Cardio-respiratory fitness	Blood pressure values	Endurance parameters
Lovell et al. (2010) [27]	75.2 ± 0.8	CET 50–70 % of VO_2max_ 30–45 min/session 3 sessions/week, 16 weeks	Training group: ↑ in VO_2max_ (from 22.6 ± 0.7 to 25.9 ± 0.9 ml/min/kg). ↔ in Control group.		
Vogel et al. (2011) [28]	70.8 ± 5.2	CET 5-min stages consisting of 4 min at VT_1_ and 1 min at 90 % of MTP 30 min/session 2 sessions/week, 9 weeks	↑ in the VO_2max_ (from 16.9 ± 3.6 to 19.7 ± 3.6 ml/min/kg) and (from 21.4 ± 5.8 to 23.3 ± 6.4 ml/min/kg), in older women and men, respectively.		↑ in the VT_1_ (from 50.1 ± 11.5 to 64.1 ± 14.4 W) and (from 79.3 ± 25.1 to 96.1 ± 29.2 W) in older men and women respectively. ↓ in HR at pre-training VT_1_ (from 112 ± 18 to 105 ± 17 bpm) and (from 109 ± 19 to 103 ± 19 bpm) in older men and women respectively. ↓ in the lactate concentrations at pre-training MTP (from 4.5 ± 1.7 to 3.5 ± 1.0 mmol) and (from 5.5 ± 1.9 to 4.4 ± 19 mmol) in older men and women respectively.
Lovell et al. (2012) [29]	75.2 ± 3.0	CET 50 to 70 % VO_2max_ 30 to 45 min/session week/week, 16 weeks	Training group: ↑ in VO_2max_ (1.9 ± 0.1 to 2.2 ± 0.1 L/min). ↔ in Control group.		
Buchner et al. (2009) [30]	75	CET 75 % of HRR 30 to 35 min/session week/week, 24 to 26 weeks	Training group: ↑ in the VO_2max_ (from 16.6 ± 4.9 to 18.1 ± 2.5 ml/min/kg). ↔ in control group.		
Babcock et al. (1994) [31]	72 ± 4.4	CET 20 to 50 % of VT_2_ 40 min/session week/week, 24 weeks	Training group: ↑ in VO_2max_ (from 1.8 ± 0.2 to 2.1 ± 0.1 L/min). ↔ in control group.		↑ in VO_2_ at VT_1_ (from 1.2 ± 0.1 to 1.5 ± 0.2 L/min). ↔ in HR at VT_1_. ↔ in control group:
Malbut et al. (2002) [32]	81 ± 2.28	CET 13–15 of RPE 13 to 20 min/session 3 to 6 sessions/week, 24 weeks	↑ in the VO_2max_ of the women’s group (from 14.5 ± 2.5 to 16.2 ± 3.1 ml/kg/min). ↔ in the men’s group.		↓ in HR at VO_2_10 in the women’s group (from 108 ± 21 to 92 ± 15 bpm). ↓ in HR at VO_2_10 in the men’s group (from 97 ± 11 to 89 ± 10 bpm).
Coker et al. (2006) [33]	74 ± 1	CET 50 or 75 % of VO_2max_ 40 min/session 4 to 5 sessions/week, 12 weeks	↑ in the VO_2max_ for both the HI and MI groups (from 1.4 ± 0.3 to 1.6 ± 0.1 L/min).		
Coker et al. (2009) [34]	71 ± 1	CET 50 or 75 % of VO_2max_ 40 min/session 4 to 5 sessions/week, 12 weeks	↑ in the VO_2max_ for both the HI and MI groups (from 1.4 ± 0.3 to 1.6 ± 0.1 L/min).		
Mangione et al. (1999) [35]	71 ± 6.9	CET 40 or 70 % HRR 25 min/session week/week, 10 week	↔ in the VO_2max_ in both high and low intensity groups.		
Harber et al. (2009) [40]	71.1	CET 60 % of P_max_ 20 to 40 min/session week/week, 12 weeks	↑ in the VO_2max_ (from 1623 ± 423 to 1856 ± 480 ml/min).		
Perini et al. (2002) [41]	73.9 ± 3.5	CET 40 to 100 % of P_max_ 60 min/session week/week, 8 weeks	↑ in the VO_2max_ (from 19.3 ± 5.2 to 22.7 ± 4.9 ml/min/kg).	↓ in resting SBP (from 167 to 140 mmHg for men and from 148 to 130 mmHg for women). ↓ in resting DBP (from 88 to 78 mmHg for men and from 80 to 72 mmHg for women). ↔ in SBP and DBP on recovery time.	
Haber et al. (1984) [42]	71.1	CET 60 % of P_max_ 20 to 40 min/session week/week, 12 weeks	↑ in the VO_2max_ (from 1623 ± 423 to 1856 ± 480 ml/min).		↑ in the maximum work load (from 111 ± 36 to 129 ± 29 W).
Temfemo et al. (2011) [43]	73 ± 5	CET HR_target_ corresponding to the VT_1_ 45 min/session week/week, 8 weeks	↑ in the VO_2max_ (from 13.2 ± 1.7 to 15.3 ± 2.1 ml/min/kg).		↑ in the VT_1_ (from 9.2 ± 1.8 to 10.8 ± 2.2 ml/min/kg).
Charifi et al. (2003) [44]	73 ± 3	CET 9*4 min 65 to 75 % VO_2max_ 9*1 min 85 to 95 % VO_2max_ 45 min/session 4 sessions/week, 14 weeks	? in the VO_2max_ (from 28.8 ± 5.9 to 32.7 ± 5.4 ml/min/kg).		
Charifi et al. (2004) [45]	73 ± 3	CET 9*4 min 65 to 75 % VO_2max_ 9*1 min 85 to 95 % VO_2max_ 45 min/session 4 sessions/week, 14 weeks	↑ in the VO_2max_ (from 28.8 ± 5.9 to 32.7 ± 5.4 ml/min/kg).		
Sial et al. (1998) [46]	74 ± 2	CET 70 to 85 % HR_max_ 30 to 45 min/session 3 to 5 sessions/week, 16 weeks	↑ in the VO_2max_ (from 1.5 ± 0.2 to 1.8 ± 0.3 l/min).		
Pichot et al. (2005) [47]	73.5 ± 4.2	CET 9*4 min 65 % HR_max_ 9*1 min 85 % HR_max_ 45 min/session 4 sessions/week, 14 weeks	↑ in the VO_2max_ (from 26.8 ± 4.4 to 31.8 ± 5.1 ml/min/kg).	↔ in SBP and DBP.	
Bell et al. (2001) [48]	77 ± 7	CET 75 to 85 % VO_2max_ 40 min/session 4 sessions/week, 9 weeks	↑ in the VO_2max_ (1.05 ± 0.17 l/min to 1.22 ± 0.22 l/min) in trained leg. ↔ in untrained leg.		
Nickel et al. (2011) [36]	71 ± 7	CET 50 % of HRR 30 min/session 1 session/day, 1 day		↓ in the SBP, 15 min after exercise compared to the non-exercise day (121 ± 12 vs. 131 ± 16 mmHg; *p* < 0.05). ↓ in the DBP, 24 h after exercise compared to the non-exercise day (72 ± 8 vs. 69 ± 7 mmHg; *p* < 0.05).	
Schocken et al. (1983) [49]	72	CET 70 to 85 % HR_max_ 25 to 30 min/session week/week, 12 weeks		↔ in SBP and DBP after training program.	↑ in the maximum work load (from 690 ± 151 to 758 ± 141 Kpm/min).
Finucane et al. (2010) [37]	71.4	CET 50 to 70 % P_max_ 60 min/session week/week, 12 weeks		↔ in SBP and DBP in training and control group.	↑ in the maximum work load (from 143.9 ± 48.9 to 167.7 ± 49.7 W). ↔ in control group.
Morris et al. (2003) [38]	70.4 ± 1.2	CET 50 and 70 % of VO_2max_ 60 min/session 1 sessions/week, 8 weeks			↓ in HR and oxygen uptake after IEx compared to CEx at the same relative intensity (*p* < 0.01).

*CET* cycle ergometer training, *VO*_*2max*_ maximum volume of oxygen, *VT*_*1*_ first ventilatory threshold, *MTP* maximal tolerated power, *HR* heart rate, *HRR* heart rate reserve, *VT*_*2*_ second ventilatory threshold, *RPE* rate of perceived effort,, *VO*_*2*_ *10* oxygen consumption of 10 ml/kg/min, *P*_*max*_ *maximal workload*, *SBP* systolic blood pressure, *DBP* systolic blood pressure, *IEx* intermittent exercise, *CEx* continuous exercise, ↑ significant improvement within group, ↓ significant decrease within group, ↔ no change within group

**Table 2:** Summary of studies that analyze the effects of cycle ergometer training on metabolic outcomes

			Outcomes		
			Metabolic outcomes		
Author, year	Mean age (years)	Protocol type	Body composition	Metabolic disorders	Endocrine function
Lovell et al. (2010) [27]	75.2 ± 0.8	CET 50–70 % of VO_2max_ 30–45 min/session 3 sessions/week, 16 weeks	Training group: ↓ in body mass (from 78 ± 4.1 to 76 ± 4.2 kg). ↓ in % of body fat (from 29.2 ± 1 to 27.7 ± 1 %). ↔ in fat-free mass. ↔ in Control group.		
Finucane et al. (2010) [37]	71.4	CET 50 to 70 % P_max_ 60 min/session 3 sessions/week, 12 weeks	Training group: ↓ in weight loss (77.2 ± 16.1 to 77.0 ± 16.2 kg). ↓ in BMI (27.4 ± 4.9 to 27.3 ± 4.8 kg/m^2^). ↓ in waist circumference (98.6 ± 14.2 to 97.8 ± 13.8 cm). ↓ in intrahepatic lipid (3.7 ± 1.8 to 2.4 ± 1.0 %). ↔ in fat-free mass. ↔ in Control group.	↓ in fasting glucose, oral glucose insulin sensitivity and HbA1c in both groups.	
Harber et al. (2009) [40]	71.1	CET 60 % of P_max_ 20 to 40 min/session 3 sessions/week, 12 weeks	↓ in fat mass (from 27.6 ± 4.3 to 26.4 ± 4.1 kg). ↓ in % of body fat (from 40.7 ± 3.4 39.8 ± 3.5 %). ↑ in fat-free mass (from 39.2 ± 1.4 to 39.6 ± 1.4 kg). ↔ in body weight and BMI.		
Coker et al. (2006) [33]	74 ± 1	CET 50 or 75 % of VO_2max_ 40 min/session 4 to 5 sessions/week, 12 weeks	↔ in body weight and in % of body in MI and HI groups.	↑ in ISGD (5.0 ± 0.6 to 6.4 ± 0.5 mg/kg FFM/min) in HI group. ↔ in MI group. ↑ in non-oxidative glucose disposal in HI group (4.6 ± 0.6 to 6.0 ± 0.8 mg/kg FFM/min). ↔ in MI group.	
Coker et al. (2009) [34]	71 ± 1	CET 50 or 75 % of VO_2max_ 40 min/session 4 to 5 sessions/week, 12 weeks	↔ in body weight, BMI, and % of fat in MI and HI groups.	↔ in plasma adiponectin in MI and HI groups.	
Lovell et al. (2012) [29]	75.2 ± 3.0	CET 50 to 70 % VO_2max_ 30 to 45 min/session 3 sessions/week, 16 weeks			Training group: ↑ in the testosterone (16.9 ± 1.5 to 19.3 ± 1.8 nmol/L). ↑ in FT concentration (58.3 ± 4.9 to 67.2 ± 5.7 pmol/L). ↔ in GH, IGF-1 and SHBG concentration. ↔ in control group.
Sial et al. (1998) [46]	74 ± 2	CET 70 to 85 % HR_max_ 30 to 45 min/session 3 to 5 sessions/week, 16 weeks	↑ in fat free mass (from 49.4 ± 4.1 to 50.4 ± 4.6 kg).	↑ in the average rate of fat oxidation (from 1.6 ± 17 to 2.21 ± 28 µmol/min). ↓ in the average rate of carbohydrate oxidation (from 3.9 ± 483 to 3.2 ± 461 µmol/min). ↓ in the glucose Ra (from 1.1 ± 69 to 1.0 ± 95 µmol/min). ↔ in Ra and FFA rate of disappearance during exercise.	
Zmuda et al. (1996) [50]	70 ± 4	CET 50 to 80 % HRR 60 min/session 1 session/day, 1 day			↑ in the serum testosterone, SHBG and total serum protein (39 %, 19 %, and 13 %, respectively; *p* < 0.01). ↑ in the FT (from 0.1 ± 0.04 to 0.2 ± 0.04). ↔ in the LH concentrations.
Perini et al. (2002) [41]	73.9 ± 3.5	CET 40 to 100 % of P_max_ 60 min/session 3 sessions/week, 8 weeks	↔ in body weight and fat mass.		

*CET* cycle ergometer training, *VO*_*2max*_ maximum volume of oxygen, *P*_*max*_ *maximal workload*, *HRR* heart rate reserve, *BMI* body mass index, *MI* moderate intensity, *HI* high intensity, *ISGD* insulin-stimulated glucose disposal, *FT* free testosterone, *GH* growth hormone, *IGF-1* insulin like growth factor-1, *SHBG* sex hormone-binding globulin, *LH* luteinizing hormone, *R*_*a*_ glycerol rate of appearance, *FFA* free fatty acid, ↑ significant improvement within group, ↓ significant decrease within group, ↔ no change within group

**Table 3:** Summary of studies that analyze the effects of cycle ergometer training on functional status

			Outcomes	
			Functional status	
Author, year	Mean age (years)	Protocol type	Muscle strength	Physical performance
Lovell et al. (2010) [27]	75.2 ± 0.8	CET 50-70 % of VO_2max_ 30–45 min/session 3 sessions/week, 16 weeks	↑ in leg strength (from 45.8 ± 7.9 to 54.0 ± 8.0 kg). ↑ in peak power (from 144 ± 5 to 168 ± 6 W). ↑ in upper leg muscle mass (from 10.2 ± 5.3 to 11.0 ± 5.4 kg). ↑ in total-leg muscle mass (from 12.04 ± 4.8 to 12.9 ± 5.1 kg).	
Lovell et al. (2012) [29]	75.2 ± 3.0	CET 50 to 70 % VO_2max_ 30 to 45 min/session 3 sessions/week, 16 weeks	↑ in peak power (148 ± 5 to 168 ± 6 W). ↑ in leg strength (45.8 ± 7.3 to 54.0 ± 8.0 kg).	
Harber et al. (2009) [40]	71.1	CET 60 % of P_max_ 20 to 40 min/session 3 sessions/week, 12 weeks	↑ in the quadriceps muscle volume (from 587 ± 55 to 654 ± 60 cm^3^). ↑ in the knee extensor power (from 199 ± 25 to 261 ± 27 cm^3^). ↑ in normalized power (from 5.8 ± 0.8 to 6.5 ± 0.8 W/cm^2^). ↑ in the knee extensor power (from 4.9 ± 0.4 to 5.9 ± 0.5 Nm/cm^2^).	
Buchner et al. (2009) [30]	75	CET 75 % of HRR 30 to 35 min/session 3 sessions/week, 24 to 26 weeks	↑ in strength at the knee extension (from 88 ± 30 to 97 ± 13 Nm). ↔ in the strength of the other muscles.	↔ in gait and balance test.
Perini et al. (2002) [41]	73.9 ± 3.5	CET 40 to 100 % of P_max_ 60 min/session 3 sessions/week, 8 weeks	↔ in the maximal isometric quadriceps muscle strength, and the maximal isometric quadriceps endurance.	
Denison et al. (2013)	71.4	CET 50 to 70 % HR_max_ 60 min/session 3 sessions/week, 12 weeks	↔ in maximum grip strength in training and control groups.	Training group: ↓ in the 6 m TUG performance (from 11.0 ± 2.0 to 10.2 ± 1.6 s) in the training group compared to control group. ↔ in 3 m walk, one-legged balance and chair rise. ↔ in control group.
Malbut et al. (2002) [32]	81 ± 2.28	CET 13–15 of RPE 13 to 20 min/session 3 to 6 sessions/week, 24 weeks	↔ in isometric knee extensor strength, isometric elbow flexor strength and lower limb extensor power.	
Mangione et al. (1999) [35]	71 ± 6.9	CET 40 or 70 % HRR 25 min/session 3 sessions/week, 10 week		↓ in the chair rise time (from 23.3 ± 9.1 to 19.1 ± 6.6 s). ↑ in the 6-min walk test (from 489.6 ± 109.1 to 533.8 ± 105.0 m).

*CET* cycle ergometer training, *VO*_*2max*_ maximum volume of oxygen, *P*_*max*_ *maximal workload*, *HR*_*max*_ maximal heart rate, *RPE* rate of perceived effort, *HRR* heart rate reserve, *TUG* Timed Up and Go test, ↑ significant improvement within group, ↓ significant decrease within group, ↔ no change within group

**Table 4:** Summary of study that analyze the effects of cycle ergometer training on cognition function

			Outcomes
Author, year	Mean age (years)	Protocol type	Cognition function
Palleschi et al. (1996) [39]	74.0 ± 1.5	CET 70 % of HR_max_ 20 min/session 3 sessions/week, 3 months	↑ in the test of attentional matrix (35.9 ± 3.8 to 43.9 ± 6.3). ↑ in the verbal span test (2.8 ± 0.6 to 3.8 ± 0.6). ↑ in the supraverbal span test (7.4 ± 0.9 to 12.6 ± 2.6). ↑ in the MMSEP (19.4 ± 1.1 to 21.7 ± 1.3).

*CET* cycle ergometer training, *HR*_*max*_ maximal heart rate, *MMSEP* mini mental state examination, ? significant improvement within group

## Results

Twenty-five controlled trial studies (12 randomized studies [27, 29–38] and 13 non-randomized studies [28, 39–50]) were identified for the review.

### Effects on cardiovascular function

#### Effects on cardio-respiratory fitness

Specific effects of cycle ergometer training (CET) on cardio-respiratory fitness were reported in eight of the 12 randomized studies [27, 29–35] (Table 1). Indeed, after a moderate endurance program on cycle ergometer, Lovell et al. [27, 29] noted a significant increase in VO_2max_ (15 and 17 % respectively, *p* < 0.05) in the active group compared to the control group. Similarly, in older patients over 70, Buchner et al. [30] reported a 9 % increase in VO_2max_ (*p* < 0.05) after 78 endurance training sessions, with no significant change in the control group. Older subjects with a lower baseline VO_2max_ showed the greatest improvement in VO_2max_ (+29 %, *p* < 0.05) after CET, as shown by Babcock et al. [31]. In a small group of older people, Malbut et al. [32] reported a 15 % increase in VO_2max_ among women, and no significant change among men, after a 24-week program of CET at 75–80 % of VO_2max_. In two small randomized studies, Coker et al. [33, 34] observed a similar increase in aerobic fitness in both moderate and intermittent exercise groups as evidenced by a respective increase of 14 % and 21 % in VO_2max_, as the result of CET. Conversely, Mangione et al. [35], reported no significant change in VO_2max_ in older patients in both high and low intensity cycle training groups.

Concerning the non-randomized studies, 11 of the 13 studies were conclusive [28, 40–48] (Table 1). A recent study by Vogel et al. [28] reported that a short-term intermittent cycling exercise program (36 min per session, 2 times per week for 9 weeks) led to a significant increase in VO_2max_ (16.6 % and 8.9 % in older women and men respectively, all *p* < 0.05). Harber et al. [40] reported a significant improvement (29 %, *p* < 0.05) in the VO_2max_ of older adults after 12 weeks of CET. In a small study, Perini et al. [41] also reported an 18 % (*p* < 0.05) increase in VO_2max_ after 8 weeks of CET. Other data suggested that CET in people over 70 resulted in an 11 % (*p* < 0.01) increase in VO_2max_ [42]. Moreover, short-term CET led to a significant 16 % (*p* < 0.001) improvement in VO_2max_ in 188 older subjects (73 ± 5 years old) [43]. Using data from two studies including older patients, Charifi et al. [44, 45] observed a 13.5 % increase in VO_2max_ (*p* < 0.05), after 56 sessions of CET. In a small study involving six older subjects (74 ± 2 years), Sial et al. [46] reported a 21 % increase in VO_2max_ (*p* < 0.01) after 16 weeks of CET at 70–85 % of maximal heart rate (HR_max_). Following 14 weeks of CET, Pichot et al. [47] observed an 18.6 % increase in the VO_2max_ (*p* < 0.01) among older adults over 70. Finally in a small study, Bell et al. [48] reported a significant 16 % increase in VO_2max_ after 36 sessions of CET at 70–85 % of VO_2max_ performed using a single leg.

According to these studies, moderate to high intensity of personalized CET, i.e., 30 min a day at least twice a week for a period of nine weeks seems to be useful for improving cardio-respiratory fitness among older adults over 70.

#### Effects on blood pressure

The benefits of CET on blood pressure in older subjects over 70 are less consistent and were reported in five studies [36, 37, 41, 47, 49] (Table 1). In a small randomized trial on healthy normotensive older patients, Nickel et al. [36] noted a significant decrease in systolic blood pressure (SBP) by 8 % after 15 min and in diastolic blood pressure (DBP) by 4 % after 24 h of CET at 50 % of heart rate reserve (HRR) in the active group compared to the control group (121 ± 12 vs. 131 ± 16 mmHg; *p* < 0.05) and (72 ± 8 vs. 69 ± 7 mmHg; *p* < 0.05), respectively. In a small pilot study including 15 older subjects, Perini et al. [41] observed a significant reduction in resting systolic (16 and 14 %, *p* = 0.001) and diastolic blood pressure (13 and 11 %, *p* = 0.004) in older men and women respectively, with no significant effects on SBP and DBP on recovery after 8 weeks of CET. However, three other studies [37, 47, 49] did not observe any significant change in SBP and DBP following CET.

Based on these studies, short-term CET seems to be associated in some extent with a significant decrease in SBP by 8–16 % and in DBP by 4–13 % in older adults over 70. This can be associated with a decrease in cardiac morbidity, stroke, and all-cause mortality.

#### Effects on endurance parameters

In this review, eight studies have shown that CET leads to significant improvement in endurance parameters of older adults over 70 [28, 31, 32, 37, 38, 42, 43, 49] (Table 1). Vogel et al. [28] reported a significant increase in the first ventilatory threshold (VT_1_) by 27.9 and 21.2 % (all, *p* < 0.05), respectively, in older men and women. Likewise, these authors reported a significant decrease in the heart rate (HR) at pre-training VT_1_ (−6.2 and −5.2 %, respectively; *p* < 0.05) as well as in lactate concentrations at pre-training maximal tolerated power (MTP) (−23.4 and −20 %, respectively; *p* < 0.05), after nine weeks of CET. In a small group of older people, Morris et al. [38] reported that at the same relative intensity (50 % or 70 % of VO_2max_), interval exercise training resulted in a significant reduction in HR and oxygen uptake as opposed to continuous exercise training (*p* < 0.01). In a small group of older adults (72 ± 4.4 years old), Babcock et al. [31] reported a 21 % (*p* < 0.05) increase in oxygen uptake (VO_2_) at VT_1_. However, they did not report any significant change in HR at VT_1_ after 72 exercise sessions of CET. In another study in older men and women, Malbut et al. [32] reported a significant decrease in HR at VO_2_10 (heart rate at an oxygen consumption of 10 ml/kg/min) by 8 % (*p* < 0.05) and 15 % (*p* < 0.01) respectively, after 24 weeks of CET. Temfemo et al. [43] reported a significant increase in VT_1_ (17 %, *p* < 0.05) in older patients after 8 weeks of CET at VT_1_. Finally, Schocken et al. [49], Haber et al. [42] and Finucane et al. [37] reported significant 10 % (*p* < 0.008), 16 and 16.5 % (*p* < 0.01) respective increases in the maximum workload, after 12 weeks of CET among older patients.

Based on these studies, short to medium-term of CET at an intensity of VT_1_ appears to be an optimal way of improving endurance parameters among older adults over 70, specifically through an increase in the oxygen uptake by 21 %, in the mean VT_1_ and maximum workload by 22 and 14 %, respectively. These improvements are important for seniors, and may contribute towards a better quality of life.

### Effects on metabolic outcomes

#### Effects on body composition

In this review, the effects of CET on body composition in older patients over 70 were evaluated in eight studies [27, 33, 34, 37, 40, 41, 46, 51] (Table 2). Specifically, a 16-week CET program at 50–70 % of VO_2max_ resulted in a significant decrease in body mass and in the percentage of body fat (−2.6 and −5 % respectively; *p* < 0.05), with no significant change in fat-free mass in older patients [27]. In 100 healthy older adults, Finucane et al. [37] reported a significant decrease in total body mass (−10 %, *p* = 0.007), BMI (−0.4 %, *p* = 0.03), in waist circumference (−0.8 %, *p* = 0.02) and in intrahepatic lipids (−54 %, *p* = 0.024) in the exercise group compared with controls, whereas the same authors reported no significant alteration in fat mass and fat-free mass in the two groups after 12 weeks at 50–70 % of maximum power (P_max_) of CET. However, there is evidence that the aforementioned exercise-induced adaptations can also occur independently of weight loss [51]. For example, Harber et al. [40] reported a significant decrease in fat mass (−3.9 %, *p* < 0.01) and in percentage body fat (−2.4 %, *p* < 0.01) with a significant increase in fat-free mass (−0.9 %, *p* < 0.01), without an overall decrease in body weight following 12-week of CET at 60 % of P_max_. Likewise, 16 weeks of CET at 70–85 % of HR_max_ in older patients improved fat-free mass by 2 % (*p* < 0.05) without an overall decrease in body weight [46]. Conversely, three studies reported no significant change in body composition after 12 weeks of CET [33, 34, 41].

These outcomes suggest that 12–16 weeks of moderate to high intensity of CET may be an appropriate way to improve the body composition among older adults over 70, specifically by a significant decrease in the mean percentage of body fat and of body mass by 3.7 and 6.3 % respectively, and particularly by maintaining free-fat mass. These data highlighted the importance of CET when designing weight management strategies among older patients over 70.

#### Effects on metabolic disorders

Specific effects of CET on metabolic disorders were reported in 4 studies [33, 34, 37, 46] (Table 2). Indeed, Sial et al. [46] observed after 16 weeks of CET, a significant increase in the average rate of fat oxidation during exercise (33 %, *p* = 0.002), and a significant decrease in the average rate of carbohydrate oxidation during exercise (−19 %, *p* = 0.003). However, these authors did not report any significant change in the glycerol rate of appearance (Ra) and in the free fatty acid (FFA) rate of disappearance during exercise, but did report a significant 11 % decrease in glucose R_a_ after the training program (*p* = 0.01) among older adults. Further, 12 weeks of CET 4–5 times per week (40 min per session) led to a significant increase in insulin-stimulated glucose disposal (ISGD) (28 %, *p* < 0.05) as well as in non-oxidative glucose disposal (NOGD) (30 %, *p* < 0.05) only in a high-intensity (HI) training group, with no significant change in the moderate-intensity (MI) training group [33].

In another study, 12 weeks of CET led to a slight (non-significant) increase in plasma adiponectin in both MI and HI training groups [34]. Conversely, Finucane et al. [37] reported no differences in fasting glucose, oral glucose insulin sensitivity and HbA1c between the training and control groups after 12 weeks of CET.

According to the above studies, short-term of CET can be in some extent an efficient strategy to prevent the metabolic disorders among older adults over 70, specifically through an increase in the fat oxidation and a decrease in the carbohydrate metabolism during exercise as well as by enhancing overall glucose disposal which have a positive effect on the regulation of glucose metabolism. This could have important clinical implications by increasing exercise capacity in this population.

#### Effects on endocrine function

Only two of the studies reviewed analyzed the effects of CET on endocrine function in older patients [29, 50] (Table 2). Zmuda et al. [50] reported a significant increase in serum testosterone, sex hormone-binding globulin (SHBG), total serum protein and the free testosterone index (39, 19, 13 and 23 % respectively; all *p* < 0.01) with no significant change in LH concentrations, during 60 min of CET at 50–80 % of HRR. After 48 sessions of CET, Lovell et al. [29] observed a significant 14 % increase in plasma concentration of testosterone, and of 15 % in free testosterone concentration (all, *p* < 0.05), with no significant change in growth hormone (GH), insulin like growth factor-1 (IGF-1) or in SHBG concentration.

According to these two reports, CET can have significant effects on the major male reproductive hormone, mainly through an improvement in the concentration of testosterone in plasma, which, in turn, may have cardiovascular protective effects in older adults over 70.

### Effects on functional status

#### Effects on muscle strength

This review shows that CET may be a sufficient stimulus to increase muscle strength and power in older patients over 70 through seven studies [27, 29, 30, 32, 40, 41] (Table 3). For example, after 48 sessions of CET at 50–70 % of VO_2max_ (30–45 min/session), Lovell et al. [27] reported a significant increase in multiple muscle strength outcomes in older adults (i.e., leg strength (18 %), leg power (12 %), upper leg muscle mass (7 %), and total leg muscle mass (7 %), all *p* < 0.05). Similarly, Lovell et al. [29] observed a significant 18 % (*p* < 0.05) increase in leg strength and a 13.5 % (*p* < 0.05) increase in peak power after 16 weeks of CET in older patients. A 12-week exercise program (cycling 3–4 times/week, 20–45 min/session at 60–80 % of HRR) led to an 11 % improvement in quadriceps muscle volume, a 23 % increase in knee extensor power and a 31 % increase in knee extensor peak isometric force, as well as a 12 % increase in normalized power and a 20 % increase in normalized force (all, *p* < 0.05) [40]. Nine months of CET three times per week (∼35 min per session) increased the strength of the knee extensor by 10 % (*p* < 0.05), with no significant change in the strength of the other muscles [30]. Conversely, Perini et al. [41] did not report any significant change in quadriceps isometric strength or endurance after 8 weeks of CET. Likewise, Denison et al. reported no significant change between training and control groups in maximum grip strength after 12 weeks of CET at 50–70 of HR_max_. Finally, Malbut et al. [32] observed no effect of CET in isometric knee extensor strength, isometric elbow flexor strength or lower limb extensor power among older patients.

To summarize the above data, CET for 20–45 min, three times a week for 12–16 weeks at 50–70 % of VO_2max_, is an efficient way of maintaining and improving muscle strength among older adults over 70, specifically through the development of leg and knee extension strength as well as the strength of the upper and total leg muscle mass.

#### Physical performance

Conflicting results have been reported concerning the effect of CET on the physical performance of older patients over 70 [30, 35] (Table 3). In a randomized controlled trial, Buchner et al. [30] did not observe any significant improvement in gait and balance test, after 24 weeks of CET. Denison et al., reported only a significant decrease in the 6 m timed up and go test (TUG) by 7 % (*p* = 0.04) in the training group compared to the control group, with no significant differences between groups in 3 m walk, eyes closed one-legged balance, and chair rise test after 12-week of CET. In contrast, ten weeks of CET led to a significant improvement in chair rise time (−22 %, *p* < 0.001) and in the 6-minute walk test (9 %, *p* < 0.001) among older frail adults [35].

According to these three reports, short to medium-term CET modestly improve physical performance of older patients over 70, specifically through a decrease in the TUG as well as in chair raise time and an increase in the 6-minute walk test. Nevertheless, further studies are needed to investigate the effects of long-term CET on the physical performance of this aged population.

### Effect on cognitive function

To our knowledge, only one study has shown that CET can help improve cognition in frail older adults over 70 (Table 4). Indeed, Palleschi et al. [39] reported a significant improvement in cognitive performance, in particular an increase in the test of attentional matrix, the verbal span test, the supraverbal span test and the mini mental state examination (22, 34, 71 and 12 % respectively; *p* < 0.0001), after 3 months of CET in a small group of older patients affected by Alzheimer’s senile dementia.

According to the outcomes of this study, CET could be promoted in older adults as a cost-effective, efficient, and viable way to reduce cognitive dysfunction, but further studies are needed to confirm these finding among older patients over 70.

## Discussion

### Principal findings

To our knowledge this is the first systematic review targeting the beneficial effects of cycle ergometer training (CET) on the health of older adults over 70.

According to the current evidence we suggest that CET is a suitable form of endurance training for promoting overall physical health among older adults over 70, particularly because it is relatively easy and safe and causes no related injuries. Further, as the upper body is less in motion in bicycle ergometer training than when the individual is walking on a treadmill, it is easier to record the vital signs and to collect blood samples especially for older frail individuals.

Overall, this systematic review included 25 published studies: 12 randomized studies and 13 non-randomized studies that analyzed the effect of CET on health outcomes among older adults over 70.

These studies suggest that CET has a positive impact on cardiovascular function through an improvement in cardiorespiratory fitness, blood pressure values and endurance parameters in older individuals over 70. Specifically, it helps control metabolic outcomes through an improvement in body composition, metabolic and endocrine disorders. CET also improves functional reserve capacity mainly by increasing muscle strength, with conflicting results concerning the physical performance. Finally, one study suggests that CET may improve also the cognitive performance in older adults over 70.

### Comparison with other studies

Although several systematic reviews have already been published on the benefits of CET for the general population, to our knowledge, very few studies have focused on health benefits for older adults over 70.

Concerning the effect of cycling on cardio-respiratory performance, functional status and metabolic outcomes, our findings are in agreement with those of a systematic review by Oja et al. [20]. These authors analyzed the health benefits of cycling in middle-aged and older people. Their findings indicated clear improvement through enhancement of cardiovascular parameters, a consistent relationship between cycling and functional status through an improvement in muscle strength and regulation of metabolic function.

Although their review illustrates the potential positive relationship between cycling and health for the general population, it was not a systematic review focusing on a group of well-defined age, and it drew on a wide range of evidence. Our review provides more details on the health benefits of CET for older adults over 70, drawing on more specific intervention studies.

Valenzuela [52] studied the effect of progressive resistance training interventions in older adults aged between 80 and 89 in nursing homes. This study reported significant improvements in muscle strength and functional performance through a progressive resistance training exercise. Despite the difference in the type of training protocol, these results are in agreement with the results of our review because they also showed an improvement functional status.

On the other hand, concerning functional status, our review disagrees with the results of a review by Giné-Garriga et al. [53], who studied the effect of exercise training on functional status in frail older adults over 65. The authors of this review did not report any consistent effect of exercise training on functional status for older adults; this can probably be explained by the wide range of protocols they reviewed.

With increasing age, there is a well-described decline in voluntary physical activity, leading to an increased risk of frailty. In the present systematic review, we restricted the inclusion criteria to older adults aged over 70, whereas Keogh et al. [54] examined the effect of dancing in healthy older adults over 60. Despite the difference in the age of subjects and the type of training program, our review agrees with the results of the above review regarding the improvement in functional status and cardiorespiratory performance.

Finally, regarding functional status, our systematic review agrees with the review by Latham et al. [55], in which these authors demonstrate the beneficial effects of progressive resistance training in adults over 60.

### Strengths and limitations of the review

The main strengths of this review are the comprehensive literary search and the inclusive approach to the types of interventions examined. We searched eight literature databases across a wide range of disciplines, while imposing as few limits as possible on our search.

The strong points of our review are first, we targeted a well-defined previously neglected population of older adults over 70, and second, we revealed robust performance outcomes.

As in all risk assessments, our review has a few limitations. First, we did not include publications that were not in English; so a language bias is possible. Second, the heterogeneity of the studies and of the training protocols made it difficult to establish precise outcomes concerning the intensity, frequency and duration of exercise training across studies. Third, the age limits we chose resulted in the exclusion of a large number of articles. However, given the current challenges of providing care for an aging population, we considered it important to select information that targeted this particular frail population.

## Conclusion

Overall, this systematic review of studies on the effect of CET on the health of older adults over 70 revealed four different types of outcomes, cardiovascular function, metabolic outcomes, functional status, and cognitive performance.

Cycle ergometer training is particularly appropriate for older adults over 70 thanks to its ability to improve cardiorespiratory fitness, blood pressure values and endurance parameters. Further, CET is associated in some extent with a regulation of metabolic outcomes through an improvement in body composition and the prevention of metabolic and endocrine disorders. It has a positive effect on functional status by increasing muscle strength and in some extent enhancing physical performance. In this connection, it is worth emphasizing that this form of exercise is not only safer, but puts less stress on the joints of older adults than other typical components of exercise programs. Finally, CET may improve also the cognitive performance.

Clinicians can now use this evidence to formulate actions to enable older adults over 70 to profit from the health benefits of CET. This will reinforce the current public health efforts to improve the health of older adults, and to help them to continue pursuing their daily activities independently.
